# Spontaneously hypertensive rats exhibit increased liver flavin monooxygenase expression and elevated plasma TMAO levels compared to normotensive and Ang II-dependent hypertensive rats

**DOI:** 10.3389/fphys.2024.1340166

**Published:** 2024-04-12

**Authors:** Marta Gawryś-Kopczyńska, Mateusz Szudzik, Emilia Samborowska, Marek Konop, Dawid Chabowski, Maksymilian Onyszkiewicz, Marcin Ufnal

**Affiliations:** ^1^ Department of Experimental Physiology and Pathophysiology, Laboratory of Centre for Preclinical Research, Medical University of Warsaw, Warsaw, Poland; ^2^ Mass Spectrometry Laboratory, Institute of Biochemistry and Biophysics, Polish Academy of Sciences, Warsaw, Poland

**Keywords:** bacterial metabolites, FMO, TMAO, TMA, cardiovascular disease

## Abstract

**Background:** Flavin monooxygenases (FMOs) are enzymes responsible for the oxidation of a broad spectrum of exogenous and endogenous amines. There is increasing evidence that trimethylamine (TMA), a compound produced by gut bacteria and also recognized as an industrial pollutant, contributes to cardiovascular diseases. FMOs convert TMA into trimethylamine oxide (TMAO), which is an emerging marker of cardiovascular risk. This study hypothesized that blood pressure phenotypes in rats might be associated with variations in the expression of FMOs.

**Methods:** The expression of FMO1, FMO3, and FMO5 was evaluated in the kidneys, liver, lungs, small intestine, and large intestine of normotensive male Wistar-Kyoto rats (WKY) and two distinct hypertensive rat models: spontaneously hypertensive rats (SHRs) and WKY rats with angiotensin II-induced hypertension (WKY-ANG). Plasma concentrations of TMA and TMAO were measured at baseline and after intravenous administration of TMA using liquid chromatography-mass spectrometry (LC-MS).

**Results:** We found that the expression of FMOs in WKY, SHR, and WKY-ANG rats was in the descending order of FMO3 > FMO1 >> FMO5. The highest expression of FMOs was observed in the liver. Notably, SHRs exhibited a significantly elevated expression of FMO3 in the liver compared to WKY and WKY-ANG rats. Additionally, the plasma TMAO/TMA ratio was significantly higher in SHRs than in WKY rats.

**Conclusion:** SHRs demonstrate enhanced expression of FMO3 and a higher plasma TMAO/TMA ratio. The variability in the expression of FMOs and the metabolism of amines might contribute to the hypertensive phenotype observed in SHRs.

## Graphical Abstract

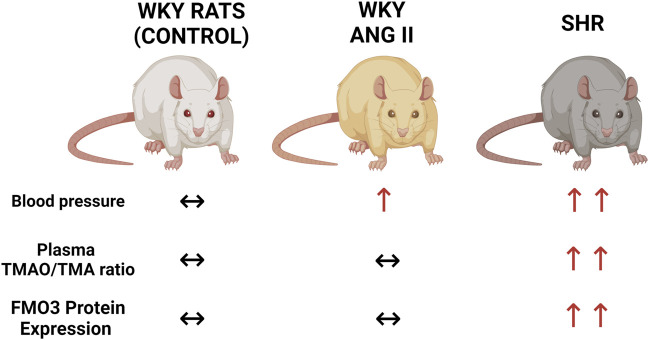

## Introduction

NADPH-dependent flavin-containing monooxygenases are a family of enzymes that catalyze the oxidation of a wide range of nitrogen-containing compounds and metabolize drugs (Eswaramoorthy et al., 2006; Phillips and Shephard, 2020). Based on the cDNA sequence, FMOs were classified into five subfamilies (FMO1 to 5) (Lawton et al., 1994; Lattard et al., 2003). Species, age, sex and tissue-dependent variability in the expression of FMOs has been described (Nagata et al., 1990; Hvattum et al., 1991; Lawton et al., 1991; Hines et al., 1994; Shehin-Johnson et al., 1995; Dolphin et al., 1996; Kawaji et al., 1997; Lattard et al., 2002; Lattard et al., 2003; Zhang and Cashman, 2006; Shimizu et al., 2011). FMOs are expressed in the liver, lungs, kidney and, to a lesser extent, in the heart, intestine and brain (Lawton et al., 1991; Bhamre et al., 1993; Kawaji et al., 1995; Bhagwat et al., 1996; Kawaji et al., 1997; Lattard et al., 2002; Novick et al., 2009).

FMO mediates N-oxygenation of tertiary amines, including vasoactive amines such as phenethylamine and tyramine, (Gut and Conney, 1993; Cashman, 1995; Mitchell et al., 1997; Cashman et al., 2004; Krueger et al., 2006). Changes in FMOs gene expression have been detected in the following diseases: trimethylaminuria (Cashman et al., 1997; Dolphin et al., 1997; Treacy et al., 1998; Akerman et al., 1999; Cashman et al., 2003), atherosclerosis (Motika et al., 2007; Shih et al., 2015), diabetes mellitus (Rouer et al., 1987; Rouer et al., 1988; Takamura et al., 2004; Toda et al., 2005), primary hemochromatosis (Muckenthaler et al., 2003; Cashman and Zhang, 2006), atrial fibrillation (Kim et al., 2003); sideroblastic anaemia (Barber et al., 2000) and in neoplastic tissues (Krueger and Williams, 2005; Fialka et al., 2008).

Trimethylamine is a gut microbiota metabolite and air pollutant originating from chemically synthetized compound used in industrial production of and an air pollutant (Pospischil et al., 2017). In mammalian organism TMA is generated by bacterial metabolism of dietary choline, betaine, and carnitine, trimethyllysine and by reduction of dietary trimethylamine N-oxide to the parent amine (Lang et al., 1998; Craciun et al., 2014; Koeth et al., 2014; Zhu et al., 2014; Hsu et al., 2019; Sun et al., 2019; Muralitharan et al., 2020). TMA is oxidized to TMAO by first-pass metabolism in the liver (Al-Waiz et al., 1987; Lin and Cashman, 1997b; Lang et al., 1998; Karoly and Rose, 2001; Krueger and Williams, 2005).

Interestingly, high TMAO concentrations has been suggested to corelate with increased cardiovascular risk (Tang et al., 2013; Qi et al., 2018). The blood TMAO level has been reported to be positively correlated with long-term mortality risk in patients with atherosclerosis, heart failure, and chronic kidney disease (Koeth et al., 2013; Tang et al., 2014; Tang et al., 2015).

However, the role of TMAO as a causative factor in cardiovascular disease is debatable as contradictory data on TMAO effects are available (Yin et al., 2015; Collins et al., 2016; Meyer et al., 2016; Huc et al., 2018; Stubbs et al., 2019; Aldana-Hernandez et al., 2020; Gawrys-Kopczynska et al., 2020; Maksymiuk et al., 2022). Previously, we have found that TMA, but not TMAO, administered intravenously IV) produced a significant hypertensive effect in normotensive rats (Jaworska et al., 2019). Furthermore, TMA after the administration was rapidly oxidized to TMAO, which was associated with a decrease in the hypertensive response (Jaworska et al., 2019).

We hypothesized that the hypertensive rat phenotype might be linked to changes in the expression and activity of FMOs. Consequently, the main aim of our study was to compare the expression of FMOs in normotensive and hypertensive rats. We carried out this experiment using two different models of hypertension: the genetic SHR model and the pharmacologically induced model using Ang II.

## Materials and methods

### Animals

All animal procedures conformed to the guidelines from Directive 2010/63/EU of the European Parliament on the protection of animals used for scientific purposes. The study was approved by the II Local Ethical Committee in Warsaw (Certificate of approval No. WAW2/082/2018). Wistar Kyoto rats (WKY) and Spontaneously Hypertensive Rats (SHR) were obtained from the Central Laboratory for Experimental Animals, Medical University of Warsaw, Poland.

Rats were housed in groups of two to three animals, in polypropylene cages with environmental enrichment, 12 h light/12 h dark cycle, temperature 22–23°C, humidity 45%–55%, food and water *ad libitum*. 12-week-old, male.

The experiments were performed on rats (WKY, n = 48) (SHR, n = 48) and (WKY-ANG, n = 48) WKY-ANG group constituted of WKY rats implanted at the age of 10 weeks with subcutaneous osmotic minipump (ALZET 2ML; Durect, Cupertino, CA). The minipumps were releasing Ang II at the rate of (0.76 pmol s−1; 0.8 ngs−1) as previously described (Zera et al., 2015). All surgical procedure were performed using general anaesthesia with ketamine 100 mg/kg body weight intraperitoneally and xylazine 10 mg/kg body weight.

### Blood pressure measurement

Before the experiment, blood pressure was recorded in rats anaesthetized with urethane (1.5 g/kg intraperitoneally, Sigma-Aldrich, Poland) via a polyurethane catheter inserted into the femoral artery. Haemodynamics were recorded using Biopac MP 160 system (Biopac Systems, Goleta, CA, United States). Blood pressure was assessed as a baseline prior to the intravenous infusion of TMA.

### Gene and protein expression

12-week-old WKY, SHR and WKY-Ang II rats were killed, tissues samples were collected and frozen immediately. Real-time PCR was used to detect FMO1, FMO3 and FMO5 gene expression in the kidney medulla, kidney cortex, liver, lungs, small intestine and colon.

### Real-time PCR

In short, about 20 mg of every tissue was homogenized on BeadBug™ microtube homogenizer (Benchmark Scientific, Inc.). Total RNA was isolated from samples according to TRI Reagent^®^ protocol. cDNA was transcribed from RNA samples according to iScript™ Reverse Transcription Supermix protocol (Bio-Rad). The qPCR mixes were prepared according to the Bio-Rad SsoAdvanced™ universal SYBR^®^ Green Supermix protocol. Amplifications were performed in a Bio-Rad CFX Connect Real-Time System under standardized conditions using commercial assays.

We used semi-quantitative analysis of PCR products to carry out with glyceraldehyde 3-phosphate dehydrogenase (PrimePCR™ SYBR^®^ Green Assay: Gapdh, Rat, qRnoCID0057018, Bio-Rad), actin (PrimePCR™ SYBR^®^ Green Assay: Actb, Rat, qRnoCID0056984, Bio-Rad), succinate dehydrogenase (PrimePCR™ SYBR^®^ Green Assay: Sdha, Rat, qRnoCID0057011, Bio-Rad) as internal references.

Genes investigated in this study were flavin containing monooxygenase 1 (PrimePCR™ SYBR^®^ Green Assay: FMO1, Rat, qRnoCID0008990, Bio-Rad), flavin containing monooxygenase 3 (PrimePCR™ SYBR^®^ Green Assay: FMO3, Rat, qRnoCID0003196, Bio-Rad) and flavin containing monooxygenase 5 (PrimePCR™ SYBR^®^ Green Assay: FMO5, Rat, qRnoCID0053250, Bio-Rad).

### Western blot

For the analysis of target proteins, total protein extracts were prepared from the, liver,. In short, frozen samples were suspended in a histidine-sucrose buffer (30 mM histidine, 250 mM sucrose, 2 mM EDTA, proteases inhibitors, PMSF, pH 7.4), homogenized, centrifuged (10,000 RCF, 10 min, 4°C). After removing the supernatant, 150 µL of lysis buffer (20 mM HEPES pH 7.4, 150 mM NaCl, 1 mM EDTA, 2% Triton-X, proteases inhibitors) was added to the pellet and resuspended by vortexing. The supernatant was separated for protein concentration analysis using a Bradford Protein Assay (Bio-Rad, Hercules, CA, United States). For all Western blot analyses, a 4× Laemmli sample buffer was added to samples. To determinate the levels of FMO1, FMO3 and FMO5 all samples were resolved by electrophoresis on 12% SDS/PAGE gels. Resolved proteins were transferred onto PVDF membranes (Bio-Rad, Hercules, CA, United States), blocked using skim milk and incubated with primary and secondary antibodies. For quantitative analysis of protein content, reactive bands were quantified relative to those of actin using a ChemiDoc MP Imaging System, Densitometric analysis was performed using Quantity One software version 4.6.8 (Bio-Rad, Hercules, CA, United States). Uncropped blots and list of antibodies are presented in Supplementary Figure S5 and Supplementary Table S1.

### Pharmacokinetics of TMA, TMA/TMAO oxidation

Twelve-week-old WKY, SHR, WKY-ANG were anaesthetized with urethane (1.5 g/kg intraperitoneally, Sigma-Aldrich, Poland) and catheterized with polyurethane catheters in femoral artery and both femoral veins.

Blood samples from femoral vein, were collected at baseline, 10 min and 20 min after the intravenous infusion of TMA at a dose of 45 μmol/kg, 135 μmol/kg or 405 μmol/kg.

Plasma concentrations of TMA and TMAO was evaluated using Waters Acquity Ultra Performance Liquid Chromatograph coupled with Waters TQ-S triple-quadrupole mass spectrometer. Samples were prepared using the derivatization technique based on Johnson’s protocol with modification (Johnson, 2008). The mass spectrometer was operated in multiple-reaction monitoring (MRM)- positive electrospray ionization (ESI+) mode for all analytes. The concentrations of analytes were calculated using calibration standard mix derived from a series of calibrator samples by spiking standard stock solutions into water. Plasma samples were compared with an obtained calibration curve.

### Statistics

The Kolmogorov-Smirnov test was used to test normality of the distribution.

To evaluate changes in pharmacokinetic data in response to treatment, baseline values were compared with post-treatment values using one-way analysis of variance (ANOVA) for repeated measures. This was followed by Tukey’s *post hoc* test for multiple comparisons to identify differences between baseline and post-dose time points. Differences between groups/series were assessed using multivariate ANOVA, followed by Tukey’s *post hoc* test or by a *t*-test, as appropriate. A two-sided *p*-value of less than 0.05 was considered statistically significant. Analyses were performed using GraphPad Prism version 8.4.3 (GraphPad Software Inc., San Diego, CA, USA). Sample size calculation for Fmo’s analysis was conducted using G*Power software version 3.1.9.7, estimating a minimum required number of animals per group to be 6. Measurements was determined based on the following assumed parameters: difference between subjects (groups) 40% population mean 10 arbitrary unit (a.u) common standard deviation 0.9, for alpha error 0.05, test power 0.8. The post hoc power analysis was performed for significant differences by utilizing the online calculator: https://clincalc.com/stats/Power.aspx (Supplementary Table S2). The analysis of false discovery rate (FDR) for FMO3 mRNA and protein expression was conducted (Supplementary Tables S3, S4).

## Results

### Blood pressure at baseline

Anaesthetized SHR (n = 17) rats and WKY-ANG (n = 14), had significantly higher mean arterial blood pressure than WKY (n = 17) 118.4 ± 1.3; 110.2 ± 0.9; 76.5 ± 1.2, respectively. SHR and WKY-ANG rats showed higher heart rate than WKY 331 ± 4, 322 ± 5 and 308 ± 4. respectively. Post-hoc test revealed significant differences only between SHR vs. WKY rats (*p* < 001).

### FMO’s mRNA expression in tissues

**FIGURE 1:**
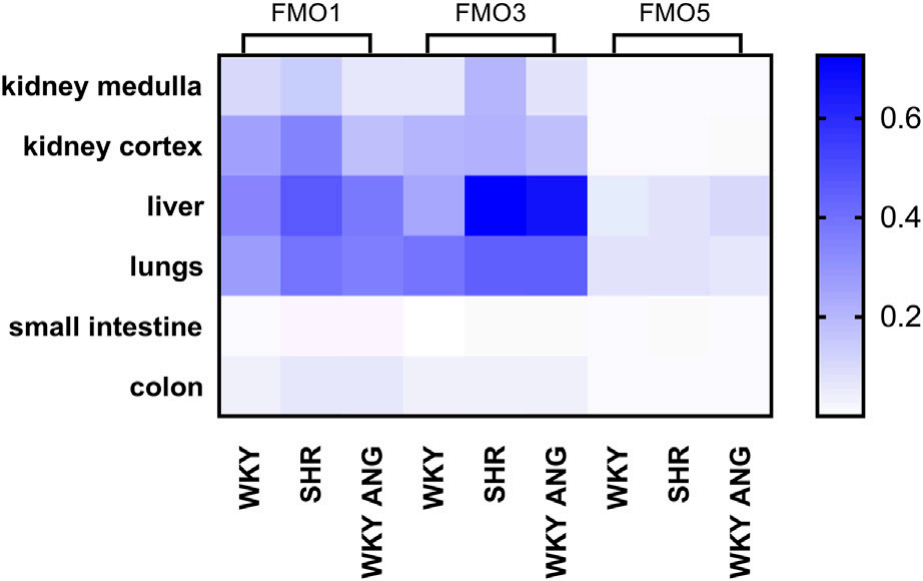
Heatmap of FMOs genes expression in WKY, SHR and WKY-ANG groups. Pattern expression peaks were found across tissues and FMOs. WKY - Wistar-Kyoto; SHR - Spontaneously Hypertensive Rats; WKY-ANG - Wistar-Kyoto with angiotensin II.

We have characterized gene and protein expressions of FMO1, FMO3 and FMO5 subfamilies, in kidney medulla, kidney cortex, liver, lungs, small intestine and colon in WKY (n = 7), SHR (n = 6), and WKY-ANG (n = 6) groups (Figure 1).

In general, all the groups, independently on tissue type, showed the gene expression of FMOs subfamilies in the following order of magnitude FMO3>FMO1>>FMO5 (Figure 1). With regard to tissue distribution of FMOs gene expression, high expression of FMOs was found in the liver, lungs and kidneys, whereas low FMOs expression was present in small intestine and colon. In relation to the liver’s most abundant mRNA FMO’s expression, we have conducted comprehensive investigations aimed at identifying the FMOs in this organ at the protein level.

### Hepatic mRNA and protein expression of FMOs

**FIGURE 2:**
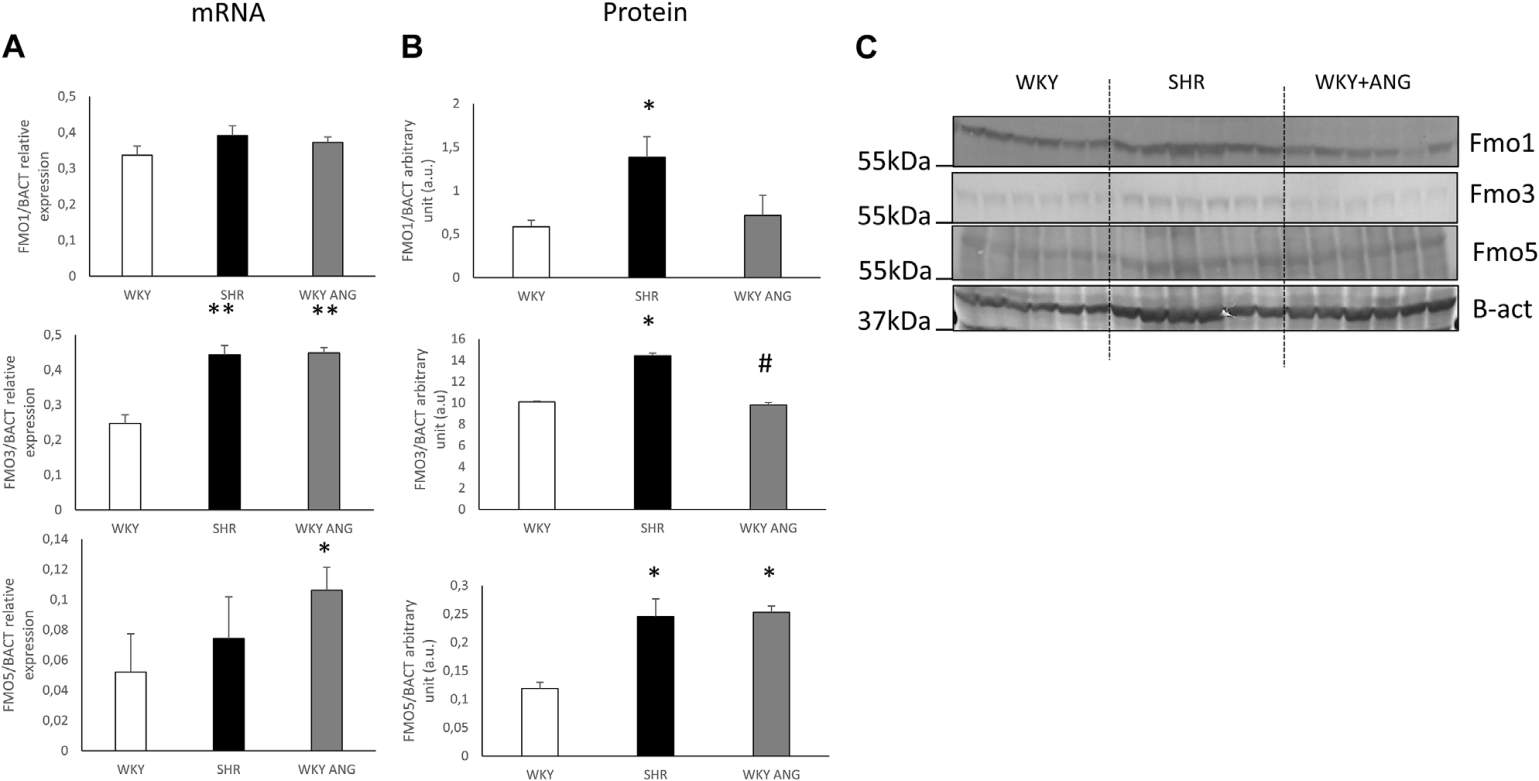
**(A)** RT-qPCR analysis of FMO1 FMO3 and FMO5 transcript levels in the liver of WKY, SHR and WKY-ANG rats (displays on histogram use arbitrary units). **(B)** FMO1, FMO3 and FMO5 protein levels in the liver examined by Western blot analysis. Beta-actin and the Ponceau-S staining were used as a reference for equal protein loading control. Quantification of the band intensity of protein expression was performed using Quantity One software The relative levels of the test proteins are plotted in arbitrary unit (means ± SD). **(C)** Representative blots of hepatic FMO’s protein of WKY, SHR and WKY-ANG rats. *p < 0.05 vs. WKY, **p < 0.01 vs. WKY #*p* < 0.05 SHR vs. WKY-ANG.

In the liver, there was notably elevated mRNA expression of FMO3 in SHR compared to WKY (*p* < 0.01), while FMO1 and FMO5 exhibited no significant differences between the two strains. Interestingly, the WKY-ANG group showed significantly higher expression levels of FMO3(*p* < 0.01) and FMO5 (*p* < 0.05) than WKY strain (Figure 2 A).

Moving to the protein level, SHR (n = 6) rats displayed significantly higher expression of all the mentioned FMOs (FMO1, FMO3, and FMO5) (*p* < 0.05) compared to WKY (n = 6). On the other hand, the WKY-ANG (n = 6) group demonstrated significantly higher expression of only FMO5 (*p* < 0.05) when compared to WKY (Figures 2B,C).

All statistical comparisons were made against WKY which was a control group in all gene and protein-based experiments.

### Pharmacokinetics of TMA/TMAO oxidation

**FIGURE 3:**
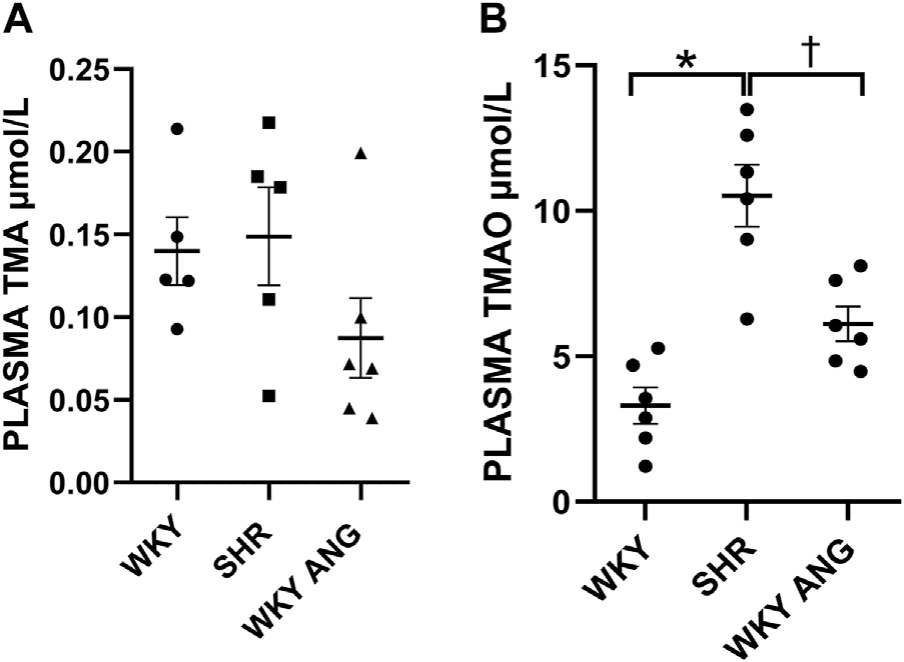
Plasma TMA **(A)** and TMAO **(B)** levels at baseline in WKY, SHR and WKY-ANG rats; *p < 0.05 SHR vs WKY, ^†^ *p* < 0.05 SHR vs WKY-ANG.

At baseline, SHR (n = 6) showed significantly higher TMAO plasma concentration than WKY (n = 6) and WKY-ANG (n = 6) 10.52 ± 0.97, 3.31 ± 0.57 and 6.11 ± 0.55 μmol/L, respectively. TMA plasma level was not significantly higher in SHR than in WKY and WKY-ANG 0.15 μmol/L ± 0.01, 0.14 μmol/L ± 0.02 and 0.09 ± 0.02 μmol/L, respectively) (Figures 3A,B).

Infusion of TMA produced a significant, dose-dependent increase in plasma TMA and TMAO in all the groups. The increase in plasma TMAO was more rapid in SHR than in the other groups (Supplementary Figures S1-S4).

**FIGURE 4:**
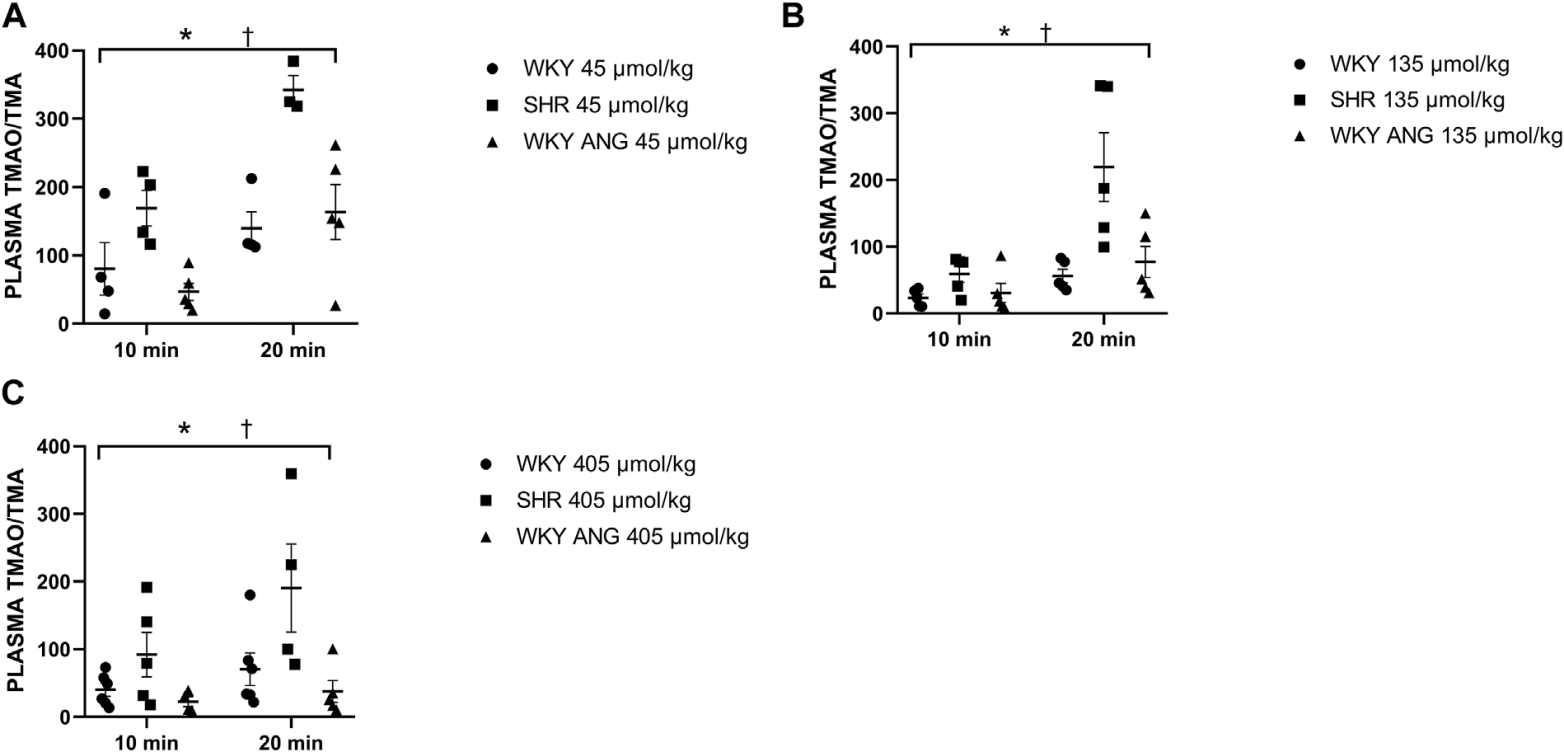
Plasma TMAO/TMA ratio in WKY, SHR and WKY-ANG rats after intravenous administration of TMA at a dose of 45 **(A)**, 135 **(B)** and 405 **(C)** μmol/kg. Two way ANOVA *p < 0.05 SHR vs WKY, ^†^ *p* < 0.05 SHR vs WKY-ANG.

SHR group showed significantly higher plasma TMAO/TMA ratio than WKY and WKY-ANG 10 min after the infusion of TMA at a dose of 45 μmol/kg, whereas 20 min after the infusion, SHR showed significantly higher plasma TMAO/TMA ratio than WKY and WKY-ANG, for all TMA doses, i.e. 45, 135 and 405 μmol/kg (Figures 4A–C).

## Discussion

The novel finding of our study is that SHRs show higher hepatic gene expression and protein levels of FMOs and more rapid oxidation of TMA to TMAO.

In the present study we evaluated two animal models of hypertension: SHRs and WKY-ANG. The SHR strain, derived from WKY rats, is the most commonly used animal model for essential hypertension in humans (Louis and Howes, 1990). SHRs begin to develop hypertension between the fourth and sixth weeks of age, and by the 10th week of life, their arterial blood pressure is 30% higher than that of WKY rats (Kokubo et al., 2005; Koga et al., 2008). Blood pressure measurements in anesthetized rats in this study revealed higher mean arterial blood pressure in both SHR and WKY-ANG rats, confirming their hypertensive phenotype.

Oxidation performed by FMOs is considered as one of important detoxifying mechanism (Sehlmeyer et al., 2010; Basaran and Can Eke, 2017). FMOs oxidize TMA as well as other amines including those exerting cardiovascular effect, for example, tyramine, phenethylamine, cys-teamine (Vrba et al., 1988), methionine and several cysteine-s-conjugates (Bull et al., 1964). Gut-bacteria derived TMA is oxidized to TMAO mostly by the FMO3 in the liver (Lang et al., 1998).

Here, we found that WKY, SHR and WKY-ANG show expression of the three subfamilies of FMO in the following order of magnitude FMO3>FMO1>>FMO5. Furthermore, we found that FMOs are expressed in the following tissues: liver, kidney, lungs, colon and intestines, with the greatest expression of FMOs was found in the liver.

In general, the most significant differences in gene and protein expression of FMOs and the pharmacokinetics of TMA were observed between the WKY and SHR, with WKY-ANG rats displaying characteristics that were a blend of both WKY and SHR strains. Specifically, compared to WKY, SHR exhibited significantly higher liver protein expression across all subfamilies of FMOs, whereas WKY-ANG rats showed an increase only in FMO5.

Importantly, the elevated expression of FMOs in SHR was linked to a more efficient and rapid oxidation of TMA to TMAO following the intravenous infusion of the amine. This was evidenced by SHRs demonstrating a significantly higher TMAO/TMA ratio after the administration of TMA in increasing doses. Lastly, SHRs also exhibited significantly higher baseline levels of TMAO, corroborating the findings of previous research (Huc et al., 2018). This study, suggests that greater oxidation of TMA to TMAO in SHRs may contribute to higher plasma TMAO levels in hypertensive rats, in addition to previously described factors such as increased gut-blood-barrier permeability to bacterial metabolites including TMA in hypertensive intestines (Jaworska et al., 2017; Drapala et al., 2020).

Some research suggest that alterations in FMOs expression are associated with several diseases including trimethylaminuria (TMAU) (Montoya Alvarez et al., 2009), diabetes mellitus (Rouer et al., 1988; Siddens et al., 2014), familial adenomatous polyposis (Cruz-Correa and Giardiello, 2003), breast (Krueger et al., 2006), prostate (Mondul et al., 2015) and colorectal cancer (Xie et al., 2012), peptic ulcer and gastro-oesophageal reflux (Chung et al., 2000) and hemochromatosis (Muckenthaler et al., 2003). Furthermore, some evidence suggests that patient with trimethylaminuria show higher blood pressure and exaggerated response to pressor amines like tyramine and phenethylamine (Forrest et al., 2001; Cashman et al., 2003), however, data are not consistent (Dolan et al., 2005; D'Angelo et al., 2013). There is also limited data on FMO3 polymorphisms and its effect on hypertension, but studies provide conflicting results (Akerman et al., 1999; Cashman et al., 2000; Cashman et al., 2003; Dolan et al., 2005; D'Angelo et al., 2013). Finally, some links between blood pressure and inactivation of biogenic amines by FMO3 (Cashman et al., 1997; Lin and Cashman, 1997; Treacy et al., 1998; Cashman et al., 2000) exist.

In the scientific literature, various models of hypertension are well-documented. For our research, we chose two models that are widely recognized and extensively used to represent human hypertension. This selection was influenced by the unique and differing etiologies of hypertension presented by these models, as well as their widespread acceptance as representative models for studying human hypertension (Jama et al., 2022). The presence of numerous underlying mechanisms driving hypertension underscores the critical need for future research to use alternative models for more comprehensive exploration.

The limitation of this study arises from its exclusive use of male rats, a decision aimed at minimizing biological variability due to hormonal fluctuations, which are known to significantly impact small experimental study outcomes. For future research, it is crucial to consider the inclusion of both sexes to ensure a more comprehensive understanding of TMA metabolism and FMOs activity in hypertensive rats. Additionally, measuring FMO expression in the heart, brain, and blood vessels would be beneficial, considering their potential impact on blood pressure and blood flow regulation within these tissues.

In conclusion, this study offers a comprehensive demonstration of the relationship between hepatic FMO expression and the oxidation of TMA to TMAO in the two animal models of hypertension. Our results indicate that hypertension in SHRs is linked to an increased expression and activity of liver FMOs. Further experimental research is necessary to clarify the role of FMOs in the pathogenesis of cardiovascular diseases. The findings from this study lay the groundwork for subsequent investigations into FMOs as a potential therapeutic target for hypertension treatment.

## Data Availability

The original contributions presented in the study are included in the article/supplementary materials, further inquiries can be directed to the corresponding author.
