# Supplementary material for: Spontaneously hypertensive rats exhibit increased liver flavin monooxygenase expression and elevated plasma TMAO levels compared to normotensive and Ang II-dependent hypertensive rats
Source: Front Physiol. 2024 Apr 12;15:1340166. doi: 10.3389/fphys.2024.1340166 (PMC11046708; doi:10.3389/fphys.2024.1340166)
Supplement: Supplementary file 1 [file DataSheet1.DOCX]

**Supplementary figure 1.**

Upper panel shows plasma TMAO level (µmol/L) before and after intravenous administration of NaCl) in WKY, SHR and WKY ANG group (*n* = 6; mean with SEM). Lower panel shows plasma TMA level (µmol/L) before and after intravenous administration of NaCl) in WKY, SHR and WKY ANG group (*n* = 6; mean with SEM).

**Supplementary figure 2.**

Upper panel shows plasma TMAO level (µmol/L) in WKY, SHR and WKY ANG group after intravenuos administation of 45 µmol/kg TMA (n=6; mean with SEM). * WKY vs SHR p=0,00732; † WKY ANG vs SHR p=0,00300. Lower panel shows plasma TMAO level (µmol/L) in WKY, SHR and WKY ANG group after intravenous administration of 45 µmol/kg TMA (n=6; mean with SEM).

SHR group plasma TMAO level was significantly higher than in WKY and WKY ANG groups (in order p=0,00732; p=0,00300).

**Supplementary figure 3.**

Upper panel shows plasma TMAO level (µmol/L) in WKY, SHR and WKY ANG group after intravenuos administation of 135 µmol/kg TMA (n=6; mean with SEM). * WKY vs SHR p=0,01677; † WKY ANG vs SHR p=0,00629. Lower panel shows plasma TMAO level (µmol/L) in WKY, SHR and WKY ANG group after intravenous administration of 135 µmol/kg TMA (n=6; mean with SEM).

SHR group plasma TMA level is significantly lower than in WKY and WKY ANG groups (in order p=0,01677; p=0,00629).

**Supplementary figure 4.**

Upper panel shows plasma TMAO level (µmol/L) in WKY, SHR and WKY ANG group after intravenuos administation of 405 µmol/kg TMA (n=6; mean with SEM). † WKY ANG vs SHR p=0,012292. Lower panel shows plasma TMAO level (µmol/L) in WKY, SHR and WKY ANG group after intravenuos administation of 405 µmol/kg TMA (n=6; mean with SEM).

SHR group plasma TMA level is significantly higher than in WKY ANG group (in order p=0,012292).

**Supplementary figure 5.**

B-ACT


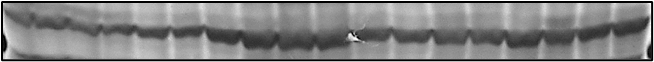


cropped blot from figure 2

37kDa


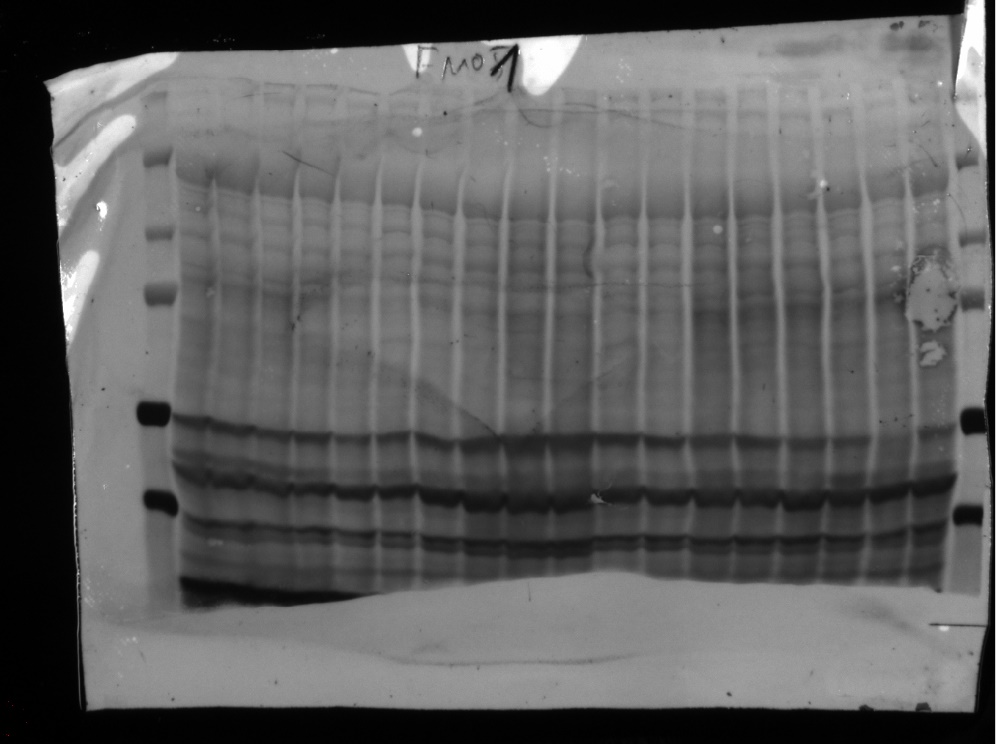


Oryginal uncropped blot

Oryginal uncropped blot


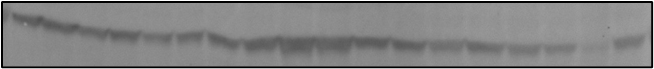


55kDa

cropped blot from figure 2


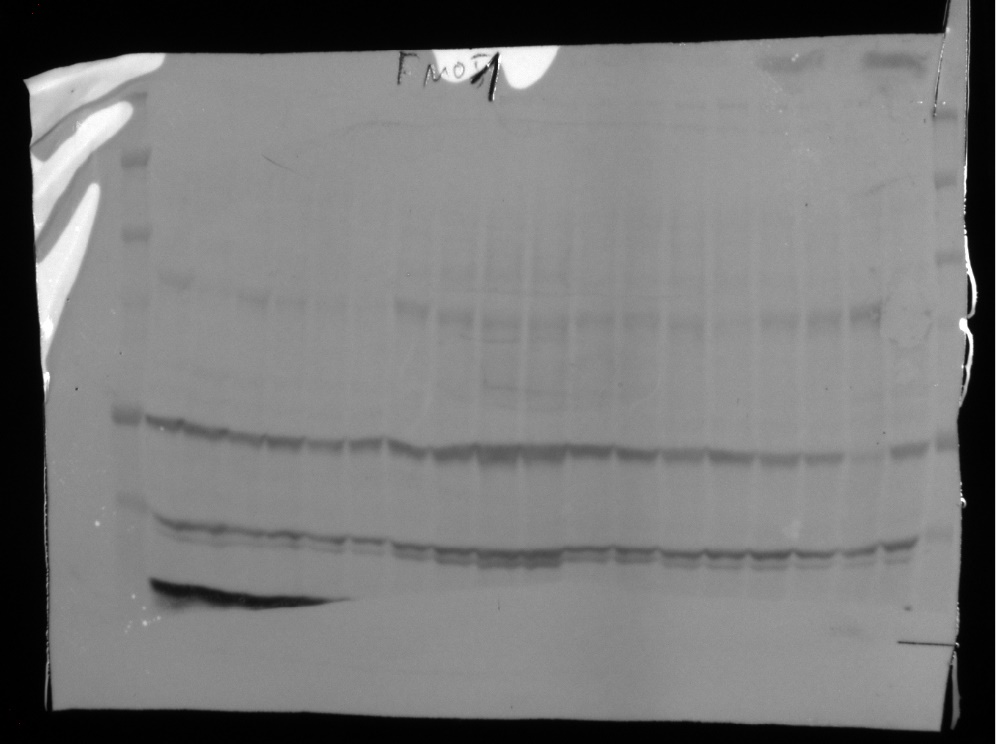


FMO1


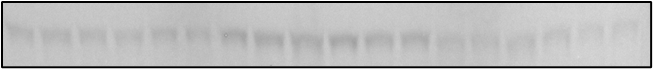


cropped blot from figure 2

55kDa


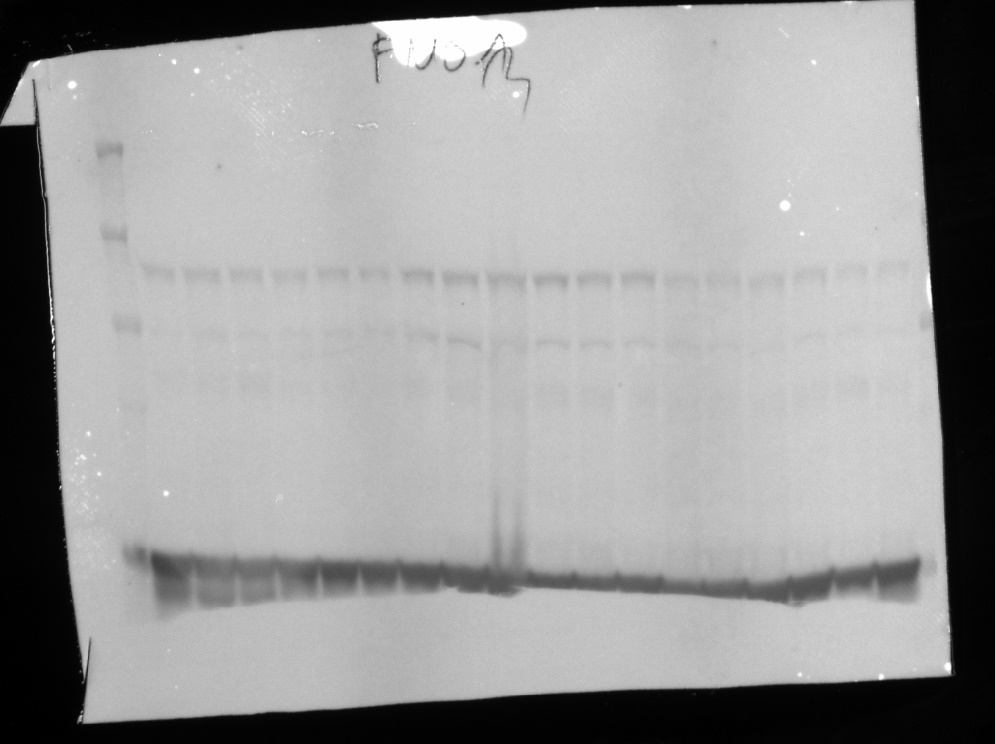


FMO3

Oryginal uncropped blot


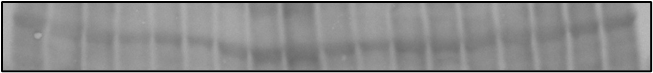


cropped blot from figure 2

55kDa


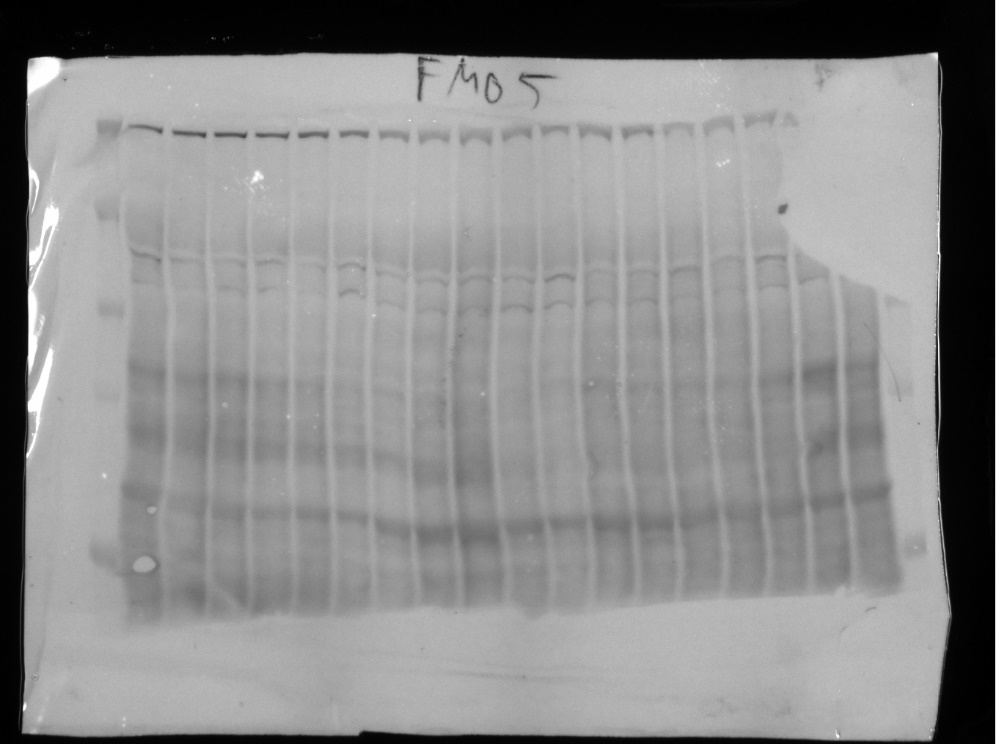


FMO5

Oryginal uncropped blot

**Supplementary table 1. List of antibodies used for Western blot analyses.**

| **Target**  **protein** | **Primary Ab** | **Dilution** | **Secondary Ab** | **Dilution** |
| --- | --- | --- | --- | --- |
| Fmo1 | Rabbit polyclonal, Abcam  ab97720 | 1:2000 | Goat anti-rabbit, Abcam  ab97048-1 | 1:10,000 |
| Fmo3 | Rabbit polyclonal, Abcam  ab126711 | 1:2000 | Goat anti-rabbit, Abcam  ab97048-1 | 1:10,000 |
| Fmo5 | Rabbit polyclonal, Invitrogen  PA5-79277 | 1:2000 | Goat anti-rabbit, Abcam  ab97048-1 | 1:10,000 |
| Beta-Actin | Goat polyclonal, Abcam  ab8229 | 1:2000 | Donkey anti-goat, Abcam  ab 97107 | 1:10,000 |

**Supplementary table 2. Post hoc power analysis conducted for confirmed significant differences.**

| Fom1 mRNA | Fmo1 protein | Fom3  mRNA | Fmo3 protein | Fom5  mRNA | Fmo5  protein | Plasma TMA | Plasma TMAO | PLASMA TMAO/TM  45 TMA uM/kg | PLASMA TMAO/TMA  135 TMA  uM/kg | PLASMA TMAO/TMA  405 TMA  uM/kg |
| --- | --- | --- | --- | --- | --- | --- | --- | --- | --- | --- |
| WKY vs SHR  N/A | WKY  vs  SHR  84.5% | WKY vs  SHR  94.7% | WKY  Vs  SHR  94.8% | WKY vs SHR  N/A | WKY  vs  SHR  94.3% | WKY  vs  SHR  N/A | WKY  Vs  SHR  100% | WKY  vs  SHR  10min  94.6%  20min  100% | WKY  vs  SHR  10min 85.7%  20min  97.6% | WKY  vs  SHR  10min  85.3%  20min  95.8% |
| WKY vs WKY ANG  N/A | WKY  vs  WKY ANG | WKY vs WKY ANG  99.7% | WKY  vs  WKY ANG  N/A | WKY vs WKY ANG  92.2% | WKY  vs  WKY ANG  100% | WKY  vs  WKY ANG  N/A | WKY  vs  WKY ANG  N/A | WKY  vs  WKY ANG  N/A | WKY  vs  WKY ANG  N/A | WKY  vs  WKY ANG  N/A |
| SHR  vs WKY ANG  N/A | SHR  Vs  WKY ANG  N/A | SHR  vs WKY ANG  N/A | SHR  Vs  WKY ANG  95.6% | SHR  vs WKY ANG  N/A | SHR  vs  WKY ANG  N/A | SHR  vs  WKY ANG  N/A | SHR  vs  WKY ANG  100% | SHR  vs  WKY ANG  10min  96.5%  20min 99.5% | SHR  vs  WKY ANG  10min  83.6%  20min  94.5% | SHR  vs  WKY ANG  10min  86.8%  20min  94.2% |

**Supplementary table 3. FDR analysis for FMO3 protein levels**

**Supplementary table 4. FDR analysis for FMO3 mRNA levels**

FMO3 mRNA levels

FDR
